# A Src-Tks5 Pathway Is Required for Neural Crest Cell Migration during Embryonic Development

**DOI:** 10.1371/journal.pone.0022499

**Published:** 2011-07-25

**Authors:** Danielle A. Murphy, Begoña Diaz, Paul A. Bromann, Jeff H. Tsai, Yasuhiko Kawakami, Jochen Maurer, Rodney A. Stewart, Juan Carlos Izpisúa-Belmonte, Sara A. Courtneidge

**Affiliations:** 1 Sanford-Burnham Medical Research Institute, La Jolla, California, United States of America; 2 Department of Pharmacology, University of California San Diego, La Jolla, California, United States of America; 3 Salk Institute for Biological Studies, La Jolla, California, United States of America; 4 Huntsman Cancer Institute, Salt Lake City, Utah, United States of America; Kings College London, United Kingdom

## Abstract

In the adult organism, cell migration is required for physiological processes such as angiogenesis and immune surveillance, as well as pathological events such as tumor metastasis. The adaptor protein and Src substrate Tks5 is necessary for cancer cell migration through extracellular matrix in vitro and tumorigenicity in vivo. However, a role for Tks5 during embryonic development, where cell migration is essential, has not been examined. We used morpholinos to reduce Tks5 expression in zebrafish embryos, and observed developmental defects, most prominently in neural crest-derived tissues such as craniofacial structures and pigmentation. The Tks5 morphant phenotype was rescued by expression of mammalian Tks5, but not by a variant of Tks5 in which the Src phosphorylation sites have been mutated. We further evaluated the role of Tks5 in neural crest cells and neural crest-derived tissues and found that loss of Tks5 impaired their ventral migration. Inhibition of Src family kinases also led to abnormal ventral patterning of neural crest cells and their derivatives. We confirmed that these effects were likely to be cell autonomous by shRNA-mediated knockdown of Tks5 in a murine neural crest stem cell line. Tks5 was required for neural crest cell migration in vitro, and both Src and Tks5 were required for the formation of actin-rich structures with similarity to podosomes. Additionally, we observed that neural crest cells formed Src-Tks5-dependent cell protrusions in 3-D culture conditions and in vivo. These results reveal an important and novel role for the Src-Tks5 pathway in neural crest cell migration during embryonic development. Furthermore, our data suggests that this pathway regulates neural crest cell migration through the generation of actin-rich pro-migratory structures, implying that similar mechanisms are used to control cell migration during embryogenesis and cancer metastasis.

## Introduction

Initiation of cell migration requires a change in cell shape to promote a pro-migratory (or mesenchymal) phenotype, coordinated by a change in actin dynamics driven by actin-associated proteins, GTPases, kinases, and the actinomyosin cytoskeletal system [1], [2], [3]. These changes enable the cell to establish contacts with, and directionally migrate through, the extracellular matrix (ECM) in response to environmental stimuli [2]. In the adult organism, cell migration is restricted to cells that are required to traverse extracellular matrices during processes such as wound healing, angiogenesis, immune surveillance, and cancer metastasis. Migration of normal cells is most prominently found during embryogenesis where cells are required to move in 3-dimensional space to pattern the embryo and generate organs and tissues. During early development, migratory cells undergo epithelial to mesenchymal transitions (EMT), which enable the generation of a mesenchymal phenotype to promote cell migration [4]. This occurs in gastrulation during convergence and extension [5] and continues during neural crest emergence [4].

Neural crest cells are highly migratory, multipotent cells that arise in the dorsal neural tube between the neural plate and non-neural ectoderm (reviewed in [6]). These cells undergo EMT to enable delamination from the neural tube and subsequent migration to distant locations. Neural crest cells differentiate into ectomesenchymal (bone and connective tissue) and non-ectomesenchymal (neural and pigment cells) derivatives (reviewed in [7]). TGFβ induces migration of neural crest cells by upregulating many transcription factors such as Foxd3, Sox10, Twist, Snail, and Slug [8], [9] and regulating attachment to the ECM [10]. It has previously been shown that migrating neural crest cells form actin-rich, dendritic-like protrusions, which probe their surroundings, and enable them to receive cues from neighboring neural crest cells or the ECM to promote directional migration [11], [12]. Interestingly, the change to a pro-migratory phenotype induced in neural crest cells through EMT and the generation of dendritic-like projections is similar to that used by invasive tumor cells during metastasis.

One protein that has been found to regulate cancer cell invasion is the Src substrate and adaptor protein, Tks5 (originally called Fish) [13]. Tks5 has an amino-terminal phox homology (PX) domain, five SH3 domains [13], [14], and two Src phosphorylation sites. Knockdown of Tks5 expression through RNA interference results in loss of protease-dependent invasion of both Src-transformed fibroblasts and human cancer cells [15], [16], [17]. Our studies have also defined a role for Tks5 in the formation of invadopodia, actin-rich membrane protrusions that coordinate cell migration with pericellular proteolysis in vitro and tumor growth in vivo [17], [18]. Additionally, the phosphorylation of Tks5 by Src regulates the actin cytoskeleton, through association with the adaptor protein Nck, suggesting a mechanism by which Tks5-dependent invadopodia regulate cell invasion [19]. Together, these studies demonstrate that a Src-Tks5 pathway plays an important role in tumor cell migration/invasion via invadopodia formation. However, a role for this pathway in a physiological context has not been described.

We examined a role for Tks5 during embryonic development by using zebrafish, *Danio rerio*. Here we show that during embryonic development, the Src-Tks5 pathway is required for the migration of neural crest cells, a highly migratory, undifferentiated, multipotent cell type. Additionally, this pathway appears to regulate neural crest migration through the formation of pro-migratory, actin-rich, protrusions in vivo, and in both 2D and 3D culture in vitro, where these protrusions show podosomal features.

## Results

### Neural crest derivatives are affected by loss of Tks5 during embryonic development

**Figure 1:**
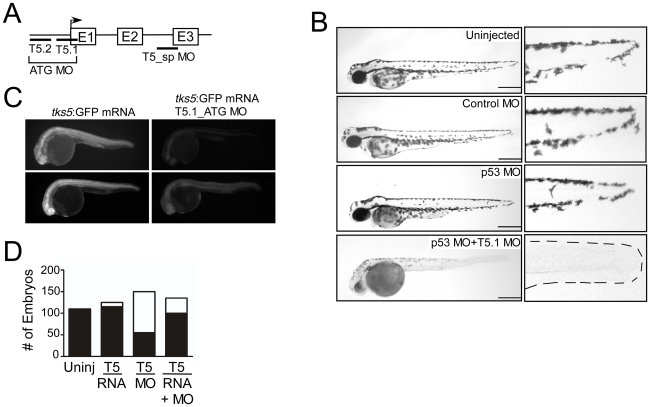
Tks5 is required for embryonic development in *Danio rerio*. (A) Schematic of Tks5 targeted morpholinos (MO) and the zebrafish Tks5 gene. (B) The morphology of Tks5 morphants (T5.1 MO+p53 MO) at 48 hpf compared to controls (Uninjected, Control MO, p53 MO). Enlarged images of the tail region show a reduction in posterior pigment cells (dashes outline tail in morphants). Scale bar represents 200 µm. (C) Tks5 MO specificity was determined by injecting embryos with either *tks5*:GFP mRNA or *tks5:*GFP mRNA together with Tks5 MO, and analyzing GFP expression. Two representative embryos from each injection are shown. (D) Quantification of murine Tks5 rescue of Tks5 morphant phenotypes. Embryos were injected as indicated in Experimental Procedures. The total number of morphants (white bars) was compared to the total number of normal embryos (black bars), and quantified as described in Materials and Methods (n = 3).

*Danio rerio* contains a single copy of a gene (designated *SH3PXD2A*) encoding a Tks5-like protein with 60% identity to the murine and human Tks5-encoding genes (data not shown). Using quantitative PCR and in situ hybridization on embryos at various stages of development, we demonstrated that Tks5 was expressed in early stages of developing embryos, and its expression increased throughout development (Figure S1A). To assess the effects of Tks5 loss on development, we microinjected one-cell stage embryos with morpholinos (MO) designed to target Tks5 (Figure 1A). At 48 hours post fertilization (hpf), Tks5 MO-injected embryos had smaller heads with small eyes, edema around the heart, and overt delay in appearance of pigment cells in the tail when compared to uninjected or 5′-mismatch (MM) control embryos (Figure 1B, S1B, S1C). Defects were also visible in the developing lateral line and in touch responses (data not shown). These phenotypes were neither rescued nor improved by co-injection with a p53 MO, which has been reported to rescue off-target effects of some MOs [20] (Figure 1B). Two non-overlapping translational Tks5 morpholinos (T5.1_ATG, T5.2_ATG, 3 ng) caused similar abnormal morphological defects (Figure S1B, data not shown). In addition, we observed the same morphological defects with a splice-blocking MO targeted to the intron 2/exon 3-splice donor/acceptor site of Tks5 (T5_sp, 5 ng) (data not shown). Reduction in Tks5 expression caused by T5_sp MO was confirmed by PCR (Figure S1D). Finally, we noted significantly diminished GFP expression from a GFP-reporter construct fused to the binding region for the Tks5_1 MO in embryos co-injected with Tks5 MO (Figure 1C). These results demonstrate the specificity of both the translational and splice-blocking morpholinos targeting Tks5, and that these Tks5 MOs can impair aspects of post-gastrulation embryonic development. For subsequent experiments, we used both T5_1 and T5_sp MO, using the generic term Tks5 MO (T5 MO), for simplicity. Details of which morpholino was used in each experiment can be found in Materials and Methods.

To confirm that Tks5 MO-induced morphological defects could be attributed to decreased Tks5 expression, we co-injected murine Tks5myc RNA (300 ng) with Tks5 MO (3 ng). Sequence differences between the species means that the zebrafish Tks5-specific morpholinos would be unable to bind to murine Tks5 RNA, and would therefore specifically silence endogenous zebrafish Tks5. Expression of murine Tks5 phenotypically rescued the Tks5 morphants, as quantified by counting the number of normal and morphant embryos within each group (Figure 1D). Tks5 expression was confirmed by immunoblotting for the myc tag in lysates of Tks5myc co-injected embryos (Figure S1E). These studies confirm that Tks5 MO-induced morphological defects are specifically attributed to the loss of Tks5 expression and that Tks5 is required for embryonic development.

**Figure 2:**
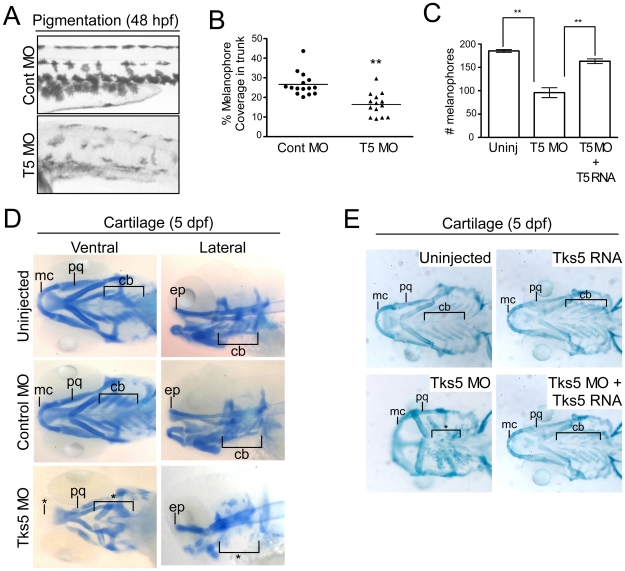
Decreased Tks5 expression results in neural crest-derived defects. (A–B) Melanophores within the trunk region above the yolk sac extension in control MO-injected and Tks5 MO-injected embryos were qualitatively (A) and quantitatively (B) analyzed. n = 15 embryos and SEM is shown by bar. p values obtained from Student's t-test. ** denotes p<0.01. (C) Melanophores present in the dorsal, ventral, and lateral pigment lines were quantified to determine degree of murine Tks5 rescue of the decreased pigmentation seen in morphants. Mean values (n = 3) and SEM are shown in graph. p values obtained from Student's t-test. ** denotes p<0.01. (D) Alcian blue staining was performed on indicated embryos to identify craniofacial structures (Meckel's cartilage (mc), palatoquadrate (pq), ceratobranchials (ch), ethmoid plate (ep)). (*) denotes missing structures. (E) Alcian blue staining was performed on indicated embryos to determine if murine Tks5 could rescue craniofacial defects seen in morphants. Structures were identified as in (D). (*) denotes missing structures.

Interestingly, the majority of defects observed in Tks5 morphants are found in neural crest-derived tissues. For example; neural crest cells are responsible for the septation of the cardiac outflow tract and for aortic arch artery development in the heart, which could be the cause of increased edema in Tks5 morphants, as well as in the generation of craniofacial structures, pigmentation, and neuronal lineages [21], [22], which are abnormal in Tks5 morphants (Figures 1B, S1B, S1C). We focused on two such tissues: pigment cells (specifically melanophores), and the craniofacial cartilage. Quantification of pigmented melanophores in the trunk region above the yolk sac extension of control and Tks5 MO-injected embryos at 48 hpf confirmed that Tks5 morphants had a significant decrease in melanophore numbers (Figures 2A,B). Co-injection of Tks5 MO with Tks5myc RNA rescued the decrease in pigmented melanophore numbers observed in the Tks5 morphants, confirming that Tks5 was required for neural crest-derived pigment cells (Figure 2C). To determine whether Tks5 MO-injected embryos had craniofacial defects, we stained uninjected, control MO-injected, and Tks5 MO-injected embryos with Alcian blue and qualitatively analyzed the cartilage structures formed. Tks5 morphants had visible malformations in the ceratobranchials, palatoquadrate, ethmoid plate, and Meckel's cartilage (Figure 2D). The majority of these defects were rescued by the co-injection of Tks5myc mRNA, demonstrating that Tks5 is also required for neural crest-derived craniofacial structures during embryonic development (Figure 2E). Together, these results indicate a specific role of Tks5 in neural crest development and proper formation of its derivatives.

### Neural crest migration in vivo

**Figure 3:**
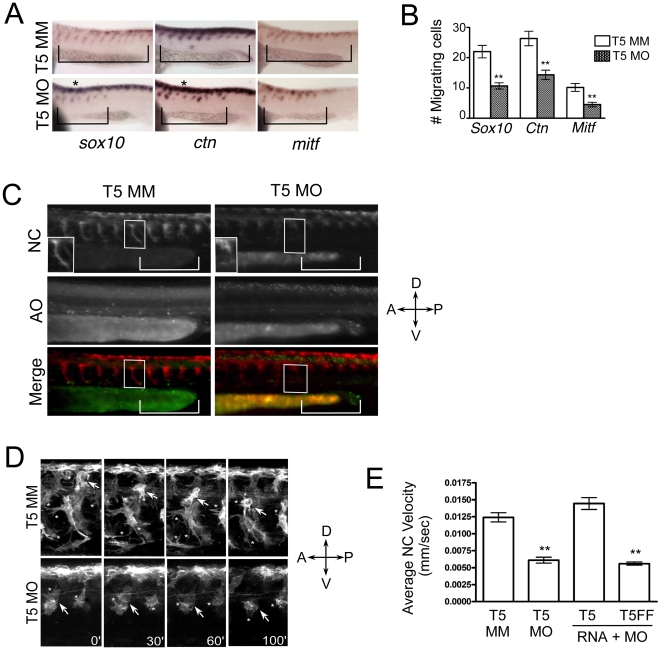
Neural crest migration in vivo requires Tks5. (A–B) Whole mount in situ hybridizations to detect neural crest cells were performed on control (Tks5 MM) and Tks5 morphant (Tks5 MO) embryos at 26 hpf. (A) Neural crest specific riboprobes against *foxd3*, *sox10*, and *crestin* (ctn) were used. Bars indicate anterior-posterior area of migrating cells. (*) indicates an increase in pre-migratory cells compared to controls (B) The number of cells migrating into the trunk region was quantified as described in Materials and Methods. Mean values (n = 18) and SEM were shown in graph. p values obtained from Student's t-test. ** denotes p<0.01. (C) Control (T5 MM) and Tks5 morphant (T5 MO) Tg(*sox10*:RFP) embryos (28 hpf) were incubated with acridine orange as a marker for apoptosis and imaged by fluorescence microscopy. NC = neural crest cells, AO = acridine orange, brackets designate similar regions of migrating NC cells, and boxes label similarly positioned individual NC cells in the control and morphant embryos (enlarged in bottom left corner of top panel). (D) Control (T5 MM) and Tks5 morphant (T5 MO) Tg(*sox10*:RFP) embryos (30 hpf) were analyzed for neural crest migration by confocal time-lapse microscopy for 1.5 hours as described in Materials and Methods. Arrows follow ventral cell migration of an individual cell over the duration. * = protrusions emanating from neural crest cells (D = dorsal, V = ventral, A = anterior, P = posterior). (E) The average velocities of individual neural crest (NC) cells for control (T5 MM)-, Tks5 MO-injected, Tks5myc RNA and Tks5 MO co-injected, or Tks5FFmyc RNA and Tks5 MO co-injected Tg(*sox10*:RFP) embryos were quantified as detailed in Materials and Methods. Mean values (n = 10) and SEM are shown. p values obtained from Student's t-test.** denotes p<0.01.

Many developmental diseases and syndromes that are associated with craniofacial dysmorphology can be attributed to defects in neural crest migration [23], [24]. Additionally, abnormal neural crest cell migration is responsible for deficiencies in pigment pattern formation in the embryo [25], [26]. Since we observed both of these defects in Tks5 morphants, we investigated whether Tks5 was required for neural crest cell migration in vivo. To ascertain this, we first conducted whole-mount in situ hybridization on control and Tks5 morphant embryos using neural crest specific probes (*sox10*, *crestin*, *mitf*). At 26 hpf, there was a decrease in the number of neural crest cells migrating dorso-ventrally in morphant embryos compared to controls (Figure 3A). This result was confirmed by quantification of *sox10*, *crestin*, and *mitf* positive cells migrating in the trunk region (Figure 3B). Additionally, we noticed a decrease in mitf-positive melanophore precursors at the posterior end of the morphant embryos, potentially contributing to the lack of melanophores in the tail region of Tks5 morphants (Figure 3A). Concomitantly, we observed a similar, if not increased number of pre-migratory, dorsally located, neural crest cells in morphant embryos compared to controls (Figure 3A, dorsal staining), suggesting that at this stage of development, interfering with Tks5 expression affected the migration or number of neural crest cells. To investigate whether the decreased number of ventrally migrating neural crest cells could be attributed to apoptosis, we stained both control and Tks5 morphant Tg(*sox10*:RFP) embryos (which have fluorescently labeled pre- and post-migratory neural crest cells [27]) with acridine orange. Tks5 morphants had an increased number of apoptotic cells on the dorsal side compared to control (Figure 3C). However, apoptotic staining did not co-localize with the majority of RFP-labeled neural crest cells (Figure 3C). Further analysis revealed minimal ventral migration of neural crest cells at the posterior end of Tks5 morphants (Figure 3C, brackets), and these ventrally migrated neural crest cells lacked extensive dendritic-like protrusions (Figure 3C, box). Together, these data suggest that abnormal patterning of neural crest cells and their derivatives is most likely attributed to migratory defects.

To look in more detail at the role of Tks5 in neural crest migration, we used confocal time-lapse microscopy to visualize Tg(*sox10*:RFP) embryos injected with either control or Tks5 MO. At 30 hpf, RFP-labeled neural crest cells in control-injected embryos could be seen migrating ventrally between the somites in the trunk region [Figure 3D, Movie S1]. In contrast, RFP-labeled neural crest cells in Tks5 morphants were present but predominantly remained on the dorsal aspect of the embryo (Figure 3D, Movie S2). Analysis of individual cells showed impaired motility and/or directionality in morphant embryos that was not observed in control embryos (Figure 3D, Movie S1, S2). In particular, there was a decrease in neural crest cell velocity in Tks5 morphant embryos compared to control (Figure 3E). This affect on neural crest migration can be attributed to loss of Tks5 since time-lapse imaging of Tg(*sox10*:RFP) embryos co-injected with both Tks5myc and Tks5 MO exhibited similar neural crest migration patterns and velocities as control embryos (Figure 3E, Movie S3). Additionally, neural crest cells in morphant embryos appeared to have a reduced number of cells elaborating protrusions during their migration as compared to control cells, suggesting a role for Tks5 in production of these protrusions during neural crest migration (Figure 3D, asterisks). These data, combined with whole-mount in situ analysis, demonstrate that Tks5 is required for neural crest cell migration during embryonic development.

### A Src-Tks5 pathway is required for NC cells and NC-derived cells during embryonic development

**Figure 4:**
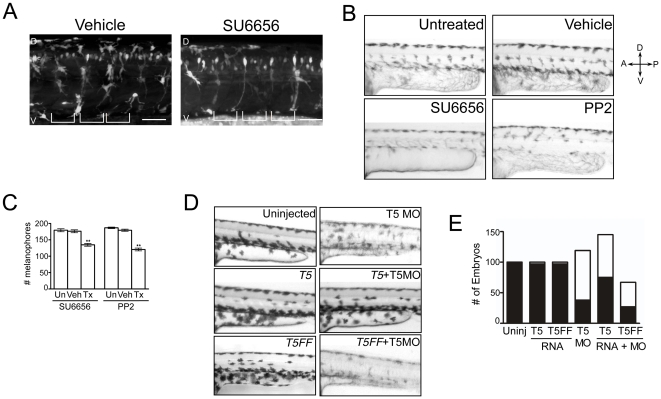
Neural crest derivatives require a Src-Tks5-dependent pathway in vivo. (A) Tg(*sox10:*RFP) embryos (8 hpf) were treated with either vehicle (DMSO) or SU6656 for 24 hours and imaged by confocal microscopy to detect neural crest cells. (D = dorsal, V = ventral). Brackets indicate the position of the somites. Scale bar represents 50 µm. (B–C) Embryos at 15 hpf were treated as indicated for 24 hours and analyzed for pigmentation defects. (B) Embryos where treatment was initiated at 15 hpf were examined for melanophore patterning in the trunk region above the yolk sac extension. (D = dorsal, V = ventral, A = anterior, P = posterior) (C) The total number of melanophores present in the dorsal and ventral pigment lines was counted for embryos within each group as described in Materials and Methods. Mean values (n = 3) and SEM were shown in graph. ** denotes p<0.01 for vehicle treated vs. SFK treated comparison. (D–E) Embryos were injected as indicated and qualitatively analyzed for defects described previously. Morpholino and RNA concentrations detailed in Materials and Methods. (E) Morphants were identified as described in Figure 1D and embryos within each group were quantified (white = morphants, black = normal).

Tks5 was first identified as a Src substrate [13], and its phosphorylation by Src is required for tumor cell migration/invasion in vitro [28]. Since we observed Tks5-dependent defects in migration of neural crest cells and neural crest-derived cells, we wanted to determine whether Src family kinases (SFKs) are also necessary for migration of neural crest cell and their derivatives in vivo. To determine this, we first treated zebrafish embryos with SU6656 and PP2 (chemically distinct pharmacological inhibitors of SFKs [29], [30]) and examined their effects on neural crest cells and neural crest-derived melanophores. Inhibitors were added after gastrulation (between 8 and 15 hpf) to specifically study the impact of SFKs on neural crest migration but circumvent the requirement for the SFKs Fyn and Yes during gastrulation of zebrafish [31], [32]. Using RFP-labeled neural crest cells in Tg(*sox10:*RFP) embryos, we found that embryos treated with SU6656 at 8 hpf appeared to have a 50% decrease in the number of ventrally migrating neural crest cells above the yolk sac extension 24 hours post-treatment. It appeared as though these cells remained primarily positioned on the dorsal side of the embryo following treatment (Figure 4A). Concurrently, there were fewer cells between somites in SU6656-treated embryos compared to vehicle treated embryos (Figure 4A). We also observed a decreased number of melanophores in the SU6656-treated embryos and these cells had abnormal patterning (Figures 4B,C). While the decreased number of melanophores may have been due to alterations in cell survival or proliferation, the abnormal patterning is likely due to migration defects, as previously stated [26]. These data suggest that the activity of one or more SFKs are required for neural crest cells and neural crest-derived cells during the segmentation-pharyngeal period of the developing embryo, and are likely to play an important role in regulating their migration.

Both Src and Tks5 affect neural crest cells and neural crest-derived cells similarly during development. Yet the presence of a Src-Tks5 pathway has yet to be observed in vivo. We therefore wanted to investigate whether Tks5 was downstream of Src in neural crest cells in zebrafish embryos. To conduct these studies, we tested a murine Tks5 construct mutated in the two Src phosphorylation sites, Y557 and Y619 [known as Tks5FFmyc], for its ability to rescue the developmental defects caused by decreased Tks5 expression (Figures 4D,E). Tks5FFmyc/Tks5MO co-injected embryos had the same, or perhaps even an increased frequency of pigmentation, craniofacial, heart, and locomotion defects when compared to embryos injected with Tks5 MO alone (Figures 4D,E, S2). This was in direct contrast to wild-type Tks5myc co-injection with Tks5 MO, which rescued the embryonic defects as previously demonstrated (Figures 4D,E, S2). Additionally, neural crest cells of Tks5FF co-injected embryos had decreased velocity and fewer cell protrusions, similar to, and perhaps more severe, than that observed in Tks5 morphants (Figure 3E, Movie S4). Together with our previous studies, these results demonstrate a requirement for both SFKs and Tks5 in neural crest cells during the segmentation-pharyngeal period of the developing embryo, and that this pathway involves, at least in part, Src-mediated phosphorylation of Tks5.

### Tks5 regulates neural crest stem cell migration and podosome formation in vitro

**Figure 5:**
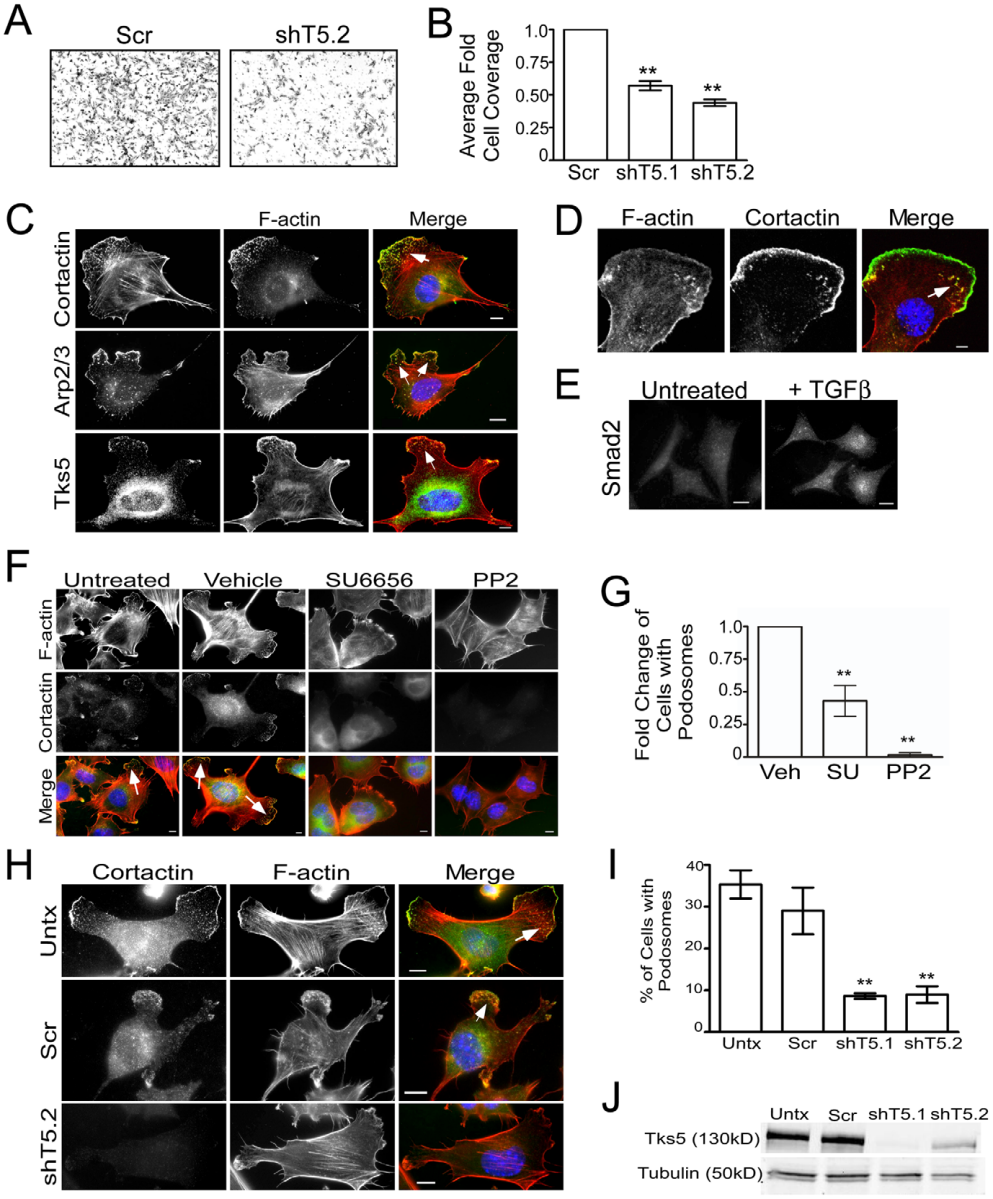
Migration of, and podosome formation in, neural crest stem cells requires Tks5. (A–B) Control (Scr) and Tks5 knockdown (shT5.1 and shT5.2) JOMA1.3 cells were exposed to a TGFβ gradient using the transwell migration assay. The number of migrating cells was qualitatively analyzed in each group (A) and quantified as described in Materials and Methods (B). Mean values (n = 3) and SEM were shown in graph. ** denotes p<0.01. (C) TGFβ-stimulated (25 ng/ml) JOMA1.3 cells were immunostained for F-actin (using phalloidin) and the podosome markers cortactin, Arp2/3, and Tks5 to identify formation of podosomes (arrows). In all cases, scale bars represent 10 µm and white arrows point to clusters of podosomes. (D) Confocal microscopy of TGFβ-stimulated JOMA1.3 cells co-stained for F-actin (using phalloidin) and cortactin. (E) JOMA 1.3 cells were treated with TGF-β and stained for SMAD2 by immunofluorescence to confirm activation of TGF-β-dependent pathways. (F–G) Vehicle (DMSO) or SFK inhibitors (SU6656 and PP2) were added to JOMA1.3 cells prior to TGF-β stimulation followed by analysis of podosome formation by immunostaining for F-actin (phalloidin) and cortactin (arrows). (G) The total number of cells with podosomes was quantified for each treatment group and analyzed as fold change of cells with podosomes compared to untreated cells. Fold change of cells was compared to vehicle treated cells. Mean values (n = 3) and SEM were shown in graph. ** denotes p<0.01 for vehicle vs. SFK treated comparison. (H–J) Tks5 was knocked-down in JOMA1.3 cells by two independent shRNA constructs [shTks5.1 (shT5.1), shTks5.2 (shT5.2)]. (H) Untreated, control (scrambled shRNA), and Tks5 knockdown cells were stimulated with TGFβ for 5 hours and stained for F-actin and cortactin to identify podosomes. (I) The percentage of cells with podosomes was quantified (as described in Materials and Methods. Mean values (n = 3) and SEM are shown. p values obtained from Student's t-test. ** denotes p<0.01. (J) Tks5 knockdown was confirmed by immunoblot analysis for Tks5 using whole cell lysates and anti-Tks5 antibody. Protein levels were normalized to tubulin.

Extracellular cues have been shown to play a role in cell migration [33]. To investigate whether the migration defects we see in Tks5 morphant embryos were cell autonomous to neural crest cells, we investigated whether Tks5 was required for neural crest cell migration in vitro by using a murine neural crest stem cell line, JOMA1.3 [34]. Since TGFβ is required for the initiation of neural crest differentiation and promotes migration [10], we placed control and Tks5 knockdown JOMA1.3 cells in transwell migration chambers containing TGFβ. We observed a two-fold reduction in migration of Tks5 knockdown cells through transwell chambers towards media containing TGFβ compared to control cells (Figures 5A–B). These data indicate that Tks5 is required for neural crest cell migration, most likely in a cell autonomous manner.

Cell migration is dependent on a shift from an adhesive to a pro-migratory phenotype, and can involve the formation of one or more actin-rich cellular protrusions (lamellipodia, filopodia, focal adhesions, podosomes, and invadopodia), which promote directional cell movement and attachment to the ECM. To investigate whether neural crest cells formed actin-based protrusions, we co-immunostained for cortactin and F-actin (using phalloidin). Under normal culture conditions, JOMA1.3 cells formed only actin stress fibers and Tks5 is cytoplasmic (Figure S3A). However, addition of either PMA or TGFβ, which are known stimulators of cell migration [35], [36], resulted in the formation of actin-rich puncta near the leading edge of the cells (Figures 5C, S3A, S3B). These actin-rich protrusions also contained Tks5 and the actin associated protein Arp2/3 (Figures 5C, S3A, S3B). Furthermore, the TGFβ-stimulated F-actin and cortactin positive puncta were co-localized in the ventral membrane as demonstrated by confocal microscopy (Figure 5D). These properties - the presence of F-actin, cortactin, Tks5 and Arp2/3, and ventral location – are used to define podosomes in other cell types. We therefore conclude that, upon cytokine stimulation, neural crest stem cells form podosomes in vitro. Finally, the induction of TGFβ responses in JOMA1.3 cells was confirmed by immunofluorescence staining for the TGFβ-regulated expression of SMAD2 in the nucleus of stimulated cells (Figure 5E).

Since Src and Tks5 are required for the formation of invadopodia in cancer cells, we wanted to determine whether these podosome structures in neural crest stem cells were also dependent on SFK activity and Tks5. SFK inhibitors prevented the formation of both PMA and TGFβ-induced podosomes in JOMA1.3 cells (Figures 5F,G, S3C, S3D). To explore whether the Src substrate Tks5 was also required, we inhibited Tks5 expression in JOMA1.3 cells via lentiviral-mediated shRNA knockdown, and then stimulated with TGFβ. Two independent, Tks5-specific shRNAs decreased TGFβ-induced podosome formation compared to control cells, and profoundly altered the cytoskeletal structure of the cells, resulting in a flattened cell appearance with decreased cortactin staining (Figures 5H–J). Similar results were observed following PMA stimulation (Figures S3E, S3F). Together, these data demonstrate that both Src and Tks5 are required for TGFβ-induced podosome formation in neural crest stem cells.

**Figure 6:**
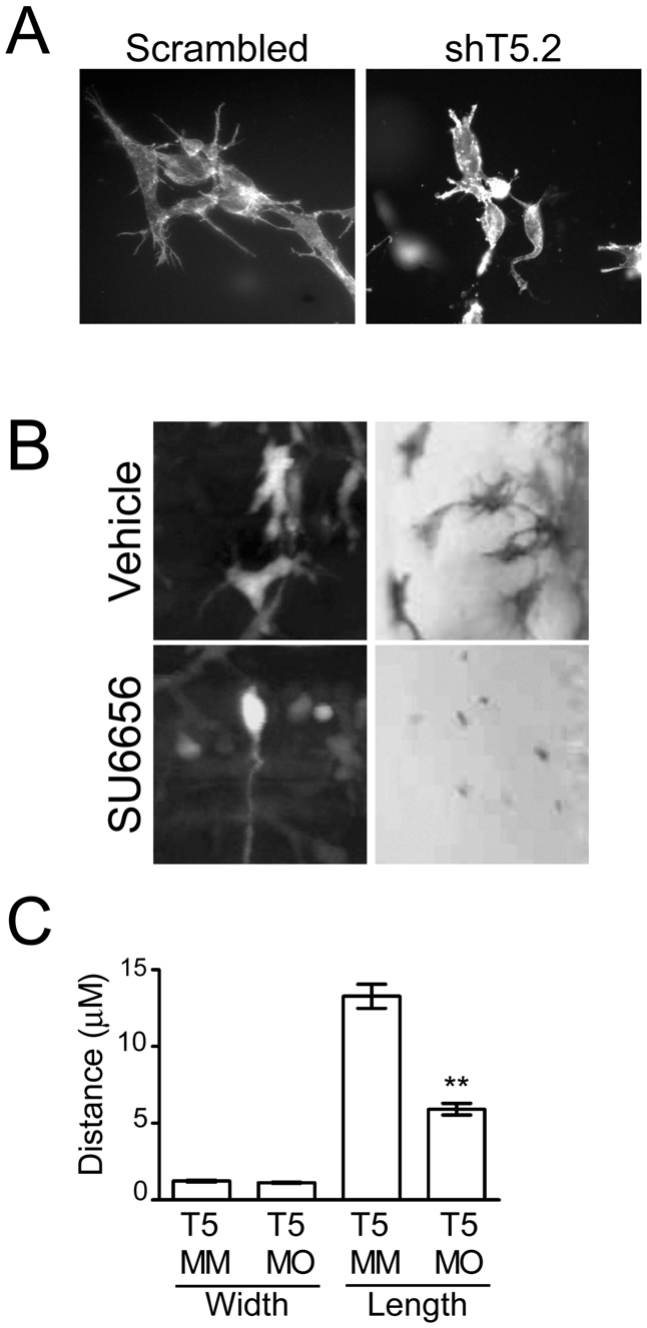
Src- and Tks5-dependent neural crest cell dendritic-like protrusions in 3-D culture and in vivo. (A) Control (scrambled) and Tks5 knocked-down (shT5.2) JOMA1.3 cells were placed in a three-dimensional collagen matrix and cultured for six days. Cells embedded in the collagen matrix were stained for F-actin (phalloidin) and analyzed for differences in cell structure (40×). (B) Neural crest cell and neural crest-derived cell protrusions were qualitatively examined by either enlarging images of neural crest cells in Tg(*sox10:*RFP) embryos obtained in 4A (left panels) or imaging melanophores in vehicle or SU6656 treated embryos at a higher magnification (23×) (right panels). (C) Control (Tks5 MM injected) and Tks5 morphant Tg(*foxd3*:GFP) embryos (30 hpf) were fixed and imaged by confocal microscopy. The width and length of protrusions was measured by Volocity software. Mean values (n = 20) and SEM are shown. p values obtained from Student's t-test. ** denotes p<0.01. (D = dorsal, V = ventral, A = anterior, P = posterior).

To investigate whether podosome-like structures are formed under more physiologically relevant conditions, we cultured JOMA1.3 in a three-dimensional (3D) collagen matrix model. We observed extensive actin-rich protrusions in control cells, but fewer and shorter protrusions in Tks5 knockdown cells (Figure 6A). Furthermore, there is a striking similarity between the Tks5-dependent protrusions formed in a 3D matrix in vitro and Tks5- and Src-dependent dendritic-like extensions emanating from the cell body of neural crest cells and derivatives in vivo (Figures 3D, 6B, 6C). We measured these extensions in control and Tks5 morphant embryos and observed a significant decrease in their length with no change in their width (Figure 6C). The width of these dendritic-like protrusions is similar to the measured width of podosomes. More importantly, the average length of these structures (10–20 µm) appeared to be more similar to podosomes (>10 µm) than those of any other pro-migratory structures such as filopodia (<10 µm). These studies suggest that a Src-Tks5-dependent pathway regulates neural crest cell migration in vivo, by regulating the ability of neural crest cells to form dendritic-like cell protrusions with similarities to podosomes.

## Discussion

We manipulated the expression of Tks5 in zebrafish using morpholino technology. Our studies revealed striking phenotypic differences between control and Tks5 morphants, implicating a role for Tks5 in multiple cell lineages during development. The majority of the defects in Tks5 morphants were in neural crest-derived organs and tissues including the heart, craniofacial structures, the lateral line and pigmentation, suggesting that Tks5 is required for neural crest cell function during embryonic development. While we cannot rule out possible minor gastrulation defects in Tks5 morphants, we believe the phenotype is not solely due to a gastrulation defect, since we observed normal extension of the embryo body and no major reduction in pre-migratory neural crest cells at early stages of development. Furthermore, we demonstrate a role for a Src-Tks5 pathway in development, and the addition of SFK inhibitors post-gastrulation also blocked dorsal-ventral distribution of neural crest cells in the trunk region.

What is the nature of the neural crest defect in Tks5 morphant zebrafish? We have provided several lines of evidence, both in vitro and in vivo, that migration of neural crest and neural crest-derived cells is impaired in the absence of Tks5. Furthermore, our in vitro studies support a cell autonomous requirement for Tks5 in neural crest cell migration in vivo. It is possible that loss of Tks5 also affects neural crest cell proliferation or differentiation. However, we believe that any impact of Tks5 on differentiation is likely to be relatively minor. For instance, in situ hybridization studies using markers for neural crest cells (foxd3 and sox10) demonstrated that there were similar, if not increased, numbers of neural crest cells on the dorsal side of Tks5 morphants at 26 hpf. Furthermore, while markers of neural crest-derived tissues, such as mitf, appeared to be reduced at 26 hpf, Tks5 morphant embryos still had abnormal posterior and ventral migration of mitf-positive cells, suggesting that those cells that have differentiated fail to migrate appropriately in the absence of Tks5. Additionally, neural crest and neural crest-derived cells were present on the dorsal aspect of morphant zebrafish, as well as zebrafish treated with SFK inhibitors. Therefore, while a potential role for Tks5 in differentiation deserves further study, we propose that the primary role of Tks5 is to participate in the directional migration of developmental cell types.

Neural crest progenitors are multipotent cells that are highly migratory during embryonic development, but the actin-cytoskeletal dynamics regulating their migration is relatively unexplored. Here we demonstrate that TGFβ stimulation of neural crest cells in vitro causes the formation of Src and Tks5-dependent podosomes, and that Tks5 and Src are required for neural crest cell migration. In vivo, migrating neural crest cells have been shown to display dendritic-like protrusions with a concentration of actin at their tips, which are dependent on Rho-kinase and myosin II for their formation [37]. Furthermore, Src activity is increased 2–7 fold in the dendrites of neural crest-derived melanocytes in vitro [38], [39]. We now find that a Src-Tks5-dependent pathway is required for both neural crest cell migration and formation of dendritic-like protrusions in neural crest cells in vivo and in vitro. Might these dendritic-like protrusions be related to podosomes? Podosomes were first discovered by their ability to mediate cell attachment to the ECM [40]. The directional migration of neural crest cells is dependent on cell-contact and their attachment to the ECM [10], [11], [12]. Therefore, it is intriguing to think that neural crest cell podosomes might regulate ECM contact during migration. Our data suggest this possibility, since there are structural and functional similarities between the Src-Tks5-dependent podosomes that we observed in 2D culture, the elongated actin-rich extensions observed in 3D culture, and the dendritic-like protrusions previously described in neural crest cells in vivo and also visualized in our study. Further analysis is clearly warranted to fully characterize and compare these protrusive structures. The podosomes and invadopodia found in adult cell types are often endowed with the ability to degrade ECM. Neural crest cells also have the capacity to degrade ECM, and they produce proteolytic enzymes, including plasminogen activator and metalloproteinases [9], [41], [42]. Additionally, electron microscopy has revealed that the basal lamina around the neural tube is incomplete during neural crest cell emigration and migration [43], [44]. Since the ECM plays an important role during development [45], with alterations in expression of collagen and the ECM proteases membrane-type 1 matrix metalloproteinase, ADAM19, and ADAM13 affecting gastrulation, neural crest cell differentiation, and migration [46], [47], [48], [49], [50], [51], it will be important to determine if the dendritic-like protrusions are involved in ECM degradation.

We recently published that mutation of the gene encoding Tks4, which is a family member of Tks5, is a cause of the human developmental disorder, Frank-Ter Haar Syndrome (FTHS), which is characterized by craniofacial and other skeletal abnormalities, as well as eye and heart defects [52]. Tks4, like Tks5, is required for invasiveness of Src-transformed cells [53], but the molecular basis of its role in FTHS is not known. The model system we describe here can be used to further dissect the roles and regulation of podosome/invadopodia-associated proteins such as Tks4 and Tks5 during embryonic development, as well as to study the genes involved in other developmental diseases that can be attributed to deficiencies in neural crest cell migration.

## Materials and Methods

### Ethics statement

All in vivo studies were reviewed and approved by an Institutional Animal Care and Use Committee (IACUC), and conducted in accordance with their guidelines. The Institutional Animal Care and Use Committee at the Salk Institute for Biological Studies approved protocol #09-029, held by Dr. Juan Carlos Izpisua-Belmonte. The Institutional Animal Care and Use Committee at the Sanford Burnham Medical Research Institute approved the protocol AUF #09-033, which is held by Dr. Sara A. Courtneidge.

### Materials

All morpholinos (MO) were designed by and obtained from GeneTools. Translational Tks5 MOs were:

T5.1 5′ ACTGCATTGTGAAAACGGAGGCTTC 3′

T5.2 5′ TTAGTGGTCAGAATAAACGGACAGG 3′

Tks5 splice (T5_sp) 5′ TGCATCTGTGGGACGACACAAGAAA 3′

p53 5′ GCGCCATTGCTTTGCAAGAATTG 3′

Tks5.1 MO was used in Figures 3, 4 and S2. Both Tks5.1 MO and Tks5_sp MO were used in Figures 1, 2 and S1. p53 MO was used at 4 ng. Tks5.1 MO and Tks5.2 MOs were used at 3.5 ng. Tks5_sp MO was used at 5 ng. pSGT-Tks5myc and pSGT-Tks5FFmyc plasmids were generated by I. Pass and F. Wen. A Tks5_1MO_GFP reporter construct was created through designing primers targeting 20 bp of Tks5_1 ATG MO and EGFP (5′ -GCC TCC GTT TTC ACA ATG CAG AGC AAG -3′) and (5′- TTA CTT GTA CAG CTC GTC CAT GGC GAG- 3′). This was placed into pCR2.1 TOPO (Invitrogen) and subcloned into pCS2 plasmid for RNA generation. *tks5:GFP* mRNA was generated using mMessage Machine SP6 kit (Ambion). *tks5:GFP* mRNA was injected at 300 ng/µl. SU6656 was synthesized by Dr. Greg Roth (SBMRI) and PP2 was obtained from EMD/Calbiochem. Antibodies used include: actin (MP Biomedicals), tubulin (Sigma), Smad2 and Hsp90 (Cell Signaling), phosphotyrosine (BD Transduction), and cortactin, Arp2/3, Tks5 and myc clone 4A6 (all from Millipore). Alexa-488 anti-mouse, Alexa-488 anti-rabbit, Alexa-568 phalloidin and Hoechst were from Invitrogen. Vectashield (Vector Labs) was used as mounting medium for all in vitro assays.

### Animals

Wild-type (AB), Tg(f*oxd3*:GFP), Tg(*sox10*:RFP), and Tg(*mlc2A:*GFP) zebrafish strains [27], [54] were maintained at 28.5°C by standard methods [55]. Staging was by morphological criteria [56]. MOs were dissolved in Danieau's buffer and injected at 2–5 ng/µl into the yolk of one-cell stage embryos; the optimal concentration was determined by dose response titrations. Embryos were either grown in 1-phenyl 2-thiourea (PTU) egg water or bleached with 0.3% hydrogen peroxide prior to staining. Fixed embryos were placed in 80% glycerol, while live embryos were placed in 1% agarose for imaging purposes.

### PCR analysis

To analyze Tks5 expression in Tks5 morphants injected with Tks5_sp, primers were designed to the Intron2/Exon3 region of Tks5 (5′-CCTTCATGCAGATACTCGAC-3′) and (5′CACGTCTCTTACATGACTTCG-3′) and RT-PCR was performed on cDNA obtained from 24 hpf embryos (50 each condition). Zebrafish Tks5 expression was analyzed by quantitative PCR using primers specific to the 5′ region of Tks5 (5′-TAAAAGTGGTGGATGTGGAG-3′). Samples were normalized to cyclophilin (5′-GTATCTCAGCATCAGGTTCG-3′) and fold increase was compared to expression in 4-cell stage embryos.

### Quantitative Analysis of Morphant Embryos

The eyes of control and Tks5 morphant embryos were examined by measuring the length and width of each embryo's eye (n>10/condition). The volume of the eye was calculated using the formula for a spheroid: [0.52*(width)^2^(length)]. Cardiac defects were examined using Tg(*mlc2A:*GFP) embryos that were either injected with control or Tks5 MO. Embryos (30hpf) were examined by fluorescence microscopy for their ability to induce heart looping during development (n>20/condition).

### Pigmentation analysis, whole-mount in situ hybridization, and staining in vivo

Control and Tks5 morphant embryos (48 hpf) were imaged by bright field microscopy to quantify melanophores in the trunk region over the yolk sac extension and analyzed as percentage of melanophore coverage by Image J software (n = 15/condition). For rescue experiments, the total number of melanophores in the ventral, dorsal, and lateral pigment lines of embryos was counted (n = 5 per condition). Embryos at 5 dpf were stained with Alcian blue as described [57] and analyzed for the presence of craniofacial structures. Neural crest specific RNA probes (*foxd3, sox10, crestin, and mitf*) were as described [57]. Migratory neural crest cells were identified as cells staining positive for *sox10*, *crestin*, or *mitf* in the trunk region between the dorsal side and yolk sac extension (between somites 5–18) of 25 hpf embryos. The total number of migrating cells was quantified for each probe (n = 6 per condition). Acridine orange (5 ug/ml, Sigma) was added to the water of control (T5 MM) and Tks5 morphant (T5 MO) Tg(*sox10*:RFP) embryos (28 hpf) for 1 hour followed by three washes in E3 medium. Anesthetized embryos were then placed in 80% glycerol and imaged by fluorescence microscopy.

### Rescue and SFK studies in vivo

Murine Tks5myc and Tks5FFmyc RNA were generated by transcribing linearized pSGT-Tks5myc and pSGT-Tks5FFmyc with mMessage Machine T7Ultra kit (Ambion). Compared to the zebrafish Tks5 MO sequence, murine Tks5myc and Tks5FFmyc RNA is homologous in only four out of 25 base pairs. Co-injection studies used 300 ng/µl Tks5myc or 500 ng/µl Tks5FFmyc with 3.5 ng Tks5 MO. Optimal concentrations were determined following dose response titrations. We characterize Tks5 morphants as possessing a decrease in pigmentation, more rounded heads, and smaller eyes. To quantify the number of morphants, 100 embryos per condition were quantified for normal or morphant phenotypes for each experiment. For rescue studies, lysates were generated from 75 embryos per condition at 24 hpf using NP40 lysis buffer. These were immunoprecipitated with anti-Myc antibody followed by SDS-PAGE immunoblot analysis for Tks5. Protein levels were normalized to Hsp90 or actin as indicated.

For SFK studies, SU6656 or PP2 were added to 0.5 ml egg water containing 1% DMSO at concentrations of 3 µM and 15 µM, respectively. These were added to wells of a 12-well plate containing 15 embryos (8 hpf or 15 hpf as indicated) per well and incubated at 28°C for 24 hours. Melanophores were quantified by counting the number in the ventral, dorsal, lateral pigment lines (10 embryos per treatment). Confocal epifluorescence microscopy was used to determine the localization and patterning of RFP-labeled neural crest cells following SU6656 treatment.

### Neural crest cells

JOMA1.3 cells are described in [34]. Phorbol-12-myristate-13-acetate (PMA) was added at 25 ng/ml for 30 minutes prior to 4% paraformaldehyde fixation. TGFβ was added at 25 ng/ml for 5 hours prior to 4% paraformaldehyde fixation. Podosomes were identified through immunofluorescence co-staining using Alexa-568 phalloidin (1∶500) and either cortactin (1∶500), Arp2/3 (1∶500), or Tks5 (1∶500). Both Alexa-488 anti-mouse and anti-rabbit secondary antibodies were used at 1∶2000. Podosome positive cells were defined as containing at least 10 positively co-stained puncta. Percentage of cells with podosomes was obtained by counting twelve fields for each condition. Localization of Smad2 was identified through immunofluorescence staining of Smad2 (1∶250).

### Tks5 and SFK studies in vitro

JOMA1.3 cells were treated with either vehicle (0.1% DMSO) or SFK inhibitors at concentrations of 2 µM (SU6656) or 10 µM (PP2) overnight prior to PMA or TGFβ stimulation and analyzed for podosome formation by immunofluorescence as previously described. Due to the variation experienced by addition of inhibitors, percentage of cells forming podosomes is normalized to vehicle for all Src inhibitor studies and analyzed as fold change in cells forming podosomes. Using similar concentration of lentivirus as control shRNA, Tks5 expression was reduced in JOMA1.3 by lentiviral shRNA vectors (shT5.1 and shT5.2). Control and Tks5 knockdown cells were treated with either PMA or TGFβ and analyzed for podosome formation. Tks5 knockdown was confirmed by obtaining protein using lysis buffer containing (50 mM Tris pH 8.0, 150 mM NaCl, 1% Tx-100, 1 mM NaF, 100 µM vanadate, 2 mM DTT), running lysates on 7.5% SDS-PAGE gels, and probing with anti-Tks5 and anti-tubulin antibodies.

### Cell migration in vitro and in vivo

Untreated or JOMA1.3 cells were infected with similar concentrations of lentivirus expressing either control (scrambled) or Tks5 knock-down (shTks5.1 or shTks5.2) and plated at 7.5×10^4^ density in transwell migration assays in the presence of TGF-β (25 ng/ml). Chamber wells were coated with a thin layer of fibronectin (0.2 mg/ml, Sigma). Five fields per condition were captured at 10× (Spot RT Acquisition and Processing Software). Area of cell coverage was quantified by Image J software. Measurements were normalized to control cells. Tg(*sox10*:RFP) embryos were injected with Tks5-mismatch MO, Tks5 MO, or co-injected with Tks5 MO and murine Tks5 RNA or murine Tks5FF RNA and imaged at 30 hpf by time-lapse laser confocal microscopy. Images were captured every ten minutes for 1.5 hours. Individual neural crest cells from the embryos described above (10 embryos/condition; 2 embryos/condition) were tracked using Volocity software by marking the location of the cell body in each single image captured for the duration of the 1.5 hours. This enabled the calculation of the velocity of each individual cell/condition using the software.

### Imaging neural crest cells in 3D in vitro and in vivo

Control (scrambled) or Tks5 knocked-down (shT5.2) JOMA1.3 cells were mixed with native collagen I (from BD) at 2 mg/ml final concentration, and plated in 8-chamber glass slides (BD Falcon) at a density of 5000 cells per chamber. Cells were cultured for 7 days and processed for fluorescence microscopy. Tg(*foxd3*:GFP) embryos were injected with either Tks5-mismatch MO or Tks5 MO, fixed at 30 hpf, and imaged by confocal microscopy. The length and width of neural crest cell protrusions were measured by Volocity software (10 cells were measured in two different embryos per condition).

### Image acquisition

All cells for podosome formation assays in vitro were imaged with a Zeiss Axioplan2 microscope and captured using an AxioCam HRm camera. JOMA1.3 were imaged at 60× magnification for identification of podosomes. Images were obtained through use of AxioVision 4.8. Zebrafish embryos were imaged using a Nikon AZ100 inverted microscope attached to a Retiga 2000R camera equipped with NIS Elements v.3 software (Nikon). Melanophore images in Figure 6 were collected using a Leica MZ16F scope attached to a Leica DFC300FX camera and acquired with Image-Pro Plus software. Confocal images were obtained using an Olympus Fluoview FV100 confocal microscope at 30°C and analyzed using FV10-ASW (Olympus) or Volocity software (Perkin Elmer). All images were processed using Image J software.

### Statistical analysis

All statistical analyses used the student's t-test, except for Figures 5B, 5G, S3D, which used Wilcoxon signed rank test.

## Supporting Information

Figure S1 **Tks5 is required for embryonic development in** ***Danio rerio*** **.** (A) Tks5 expression levels were examined throughout embryonic development in zebrafish by quantitative PCR. Levels were normalized to cyclophilin and fold increase was compared to 4-cell embryonic stage (set at 1). (B) The eye volume (µm^3^) of control (T5 MM) and Tks5 morphants (T5.1 MO and T5_sp) at 48 hpf was measured as detailed in the Materials and Methods section. Mean values (n = 12 and n = 17, respectively) and SEM are shown. p values obtained from Student's t-test.** denotes p<0.01. (C) Tg (*mlc2:*GFP) embryos (30hpf) uninjected or injected with either control (T5 MM) or Tks5 morpholinos (T5.1 MO and T5_sp) were examined for the ability to induce heart looping by fluorescence microscopy. Embryos in each group were quantified for the presence of a linear or looped heart. (D) RT-PCR analysis using exon 3 specific primers of uninjected and Tks5_sp MO-injected embryos at 24 hpf. (E) Lysates were obtained from indicated embryos at 24 hpf and immunoprecipitated with anti-Myc antibody followed by immunoblotting for Tks5. The blot was re-probed with anti-Hsp90 antibody for loading controls.
(TIF)

Figure S2 **Src phosphorylation of Tks5 affects neural crest-derived cells.** Lysates were obtained from indicated embryos (24 hpf) as described in Experimental Procedures. Tks5myc and Tks5FFmyc expression was detected by immunoprecipitation for Myc and immunoblotting for Tks5. Loading was normalized to actin.
(TIF)

Figure S3 **Neural crest stem cells form podosomes.** (A) JOMA1.3 cells were stained for F-actin (phalloidin) and either cortactin or Tks5 in the presence and absence of PMA (25 ng/ml) to identify formation of podosomes (arrow). (B) PMA-stimulated (25 ng/ml) JOMA1.3 cells were immunostained for F-actin (using phalloidin) and the podosome markers cortactin, Arp2/3, and Tks5. (C–D) Vehicle (DMSO) or SFK inhibitors (SU6656 and PP2) were added to JOMA1.3 cells prior to PMA stimulation. (C) Analysis of podosome formation was conducted by immunostaining for F-actin (phalloidin) and cortactin (arrows). (D) The total number of cells with podosomes was quantified for each treatment group and analyzed as fold change of cells with podosomes compared to untreated cells. Mean values (n = 3) and SEM were shown in graph. p values obtained from Student's t-test. * denotes p<0.05 for untreated vs. SFK treated comparison; ** denotes p<0.01 for untreated vs. SFK treated comparison. (E–F) PMA-treated control (uninfected and scrambled shRNA) and Tks5 knockdown (shT5.1, and shT5.2) cells were stained for F-actin (phalloidin) and cortactin to identify formation of podosomes. (F) Percentage of cells possessing podosomes was calculated as previously described. Mean values (n = 3) and SEM are shown in graph. ** denotes p<0.01 for scrambled versus shT5.1 and shT5.2. p values obtained from Student's t-test. In all cases, the white arrows point to clusters of podosomes.
(TIF)

Movie S1 **Neural crest cell migration in control MO-injected Tg(** ***sox10:*** **RFP) embryos at 30 hpf.** Z-projected time-lapse images from laser confocal microscopy (FV-1000, Olympus) of control MO-injected Tg(*sox10*:RFP) embryos at 30 hpf for 1.5 hours (1 frame/10 min) showed ventral migration of neural crest cells between somites towards the yolk sac extension. Also observed was the generation of cell protrusions emanating from the neural crest cells. Scale bars represent 30 µm. See Figure 3 for still images and quantification of average neural crest cell velocity.
(AVI)

Movie S2 **Reduced neural crest cell migration in Tks5 MO-injected Tg(** ***sox10:*** **RFP) embryos at 30 hpf.** Z-projected time-lapse images from laser confocal microscopy (FV-1000, Olympus) of Tks5 MO-injected Tg(*sox10:*RFP) embryos at 30 hpf for 1.5 hours (1 frame/10 min) showed a decreased ability of neural crest cells to migrate ventrally between somites towards the yolk sac extension. Cell protrusions were not prominently seen and appeared shorter in length. Scale bars represent 30 µm. See Figure 3 for still images and quantification of average neural crest cell velocity..
(AVI)

Movie S3 **Neural crest cell migration in Tks5myc RNA and Tks5 MO- co-injected Tg(** ***sox10:*** **RFP) embryos at 30 hpf.** Z-projected time-lapse images from laser confocal microscopy (FV-1000, Olympus) of Tks5myc RNA and Tks5 MO co-injected Tg(*sox10*:RFP) embryos at 30 hpf for 1.5 hours (1 frame/10 min) showed ventral migration of neural crest cells between somites towards the yolk sac extension similar to control-injected embryos (Movie S1). Scale bars represent 30 µm. See Figure 3 for quantification of average neural crest cell velocity.
(AVI)

Movie S4 **Reduced neural crest cell migration in Tks5FF RNA and Tks5 MO-co-injected Tg(** ***sox10:*** **RFP) embryos at 30 hpf.** Z-projected time-lapse images from laser confocal microscopy (FV-1000, Olympus) of Tks5FF RNA and Tks5 MO-co-injected Tg(*sox10:*RFP) embryos at 30 hpf for 1.3 hours (1 frame/10 min) showed a decreased ability of neural crest cells to migrate ventrally between somites towards the yolk sac extension. Cell protrusions were not prominently seen and appeared shorter in length. Scale bars represent 30 µm. See Figure 3 for quantification of average neural crest cell velocity.
(AVI)

## References

[1] Block MR, Badowski C, Millon-Fremillon A, Bouvard D, Bouin AP (2008). Podosome-type adhesions and focal adhesions, so alike yet so different.. Eur J Cell Biol.

[2] Friedl P, Wolf K (2010). Plasticity of cell migration: a multiscale tuning model.. J Cell Biol.

[3] Petrie RJ, Doyle AD, Yamada KM (2009). Random versus directionally persistent cell migration.. Nat Rev Mol Cell Biol.

[4] Acloque H, Adams MS, Fishwick K, Bronner-Fraser M, Nieto MA (2009). Epithelial-mesenchymal transitions: the importance of changing cell state in development and disease.. J Clin Invest.

[5] Yin C, Ciruna B, Solnica-Krezel L (2009). Convergence and extension movements during vertebrate gastrulation.. Curr Top Dev Biol.

[6] Steventon B, Carmona-Fontaine C, Mayor R (2005). Genetic network during neural crest induction: from cell specification to cell survival.. Semin Cell Dev Biol.

[7] Sauka-Spengler T, Bronner-Fraser M (2006). Development and evolution of the migratory neural crest: a gene regulatory perspective.. Curr Opin Genet Dev.

[8] Le Douarin NM, Kalcheim Chaya (1999). The Neural Crest.

[9] Sauka-Spengler T, Bronner-Fraser M (2008). A gene regulatory network orchestrates neural crest formation.. Nat Rev Mol Cell Biol.

[10] Delannet M, Duband JL (1992). Transforming growth factor-beta control of cell-substratum adhesion during avian neural crest cell emigration in vitro.. Development.

[11] Carmona-Fontaine C, Matthews HK, Kuriyama S, Moreno M, Dunn GA (2008). Contact inhibition of locomotion in vivo controls neural crest directional migration.. Nature.

[12] Jesuthasan S (1996). Contact inhibition/collapse and pathfinding of neural crest cells in the zebrafish trunk.. Development.

[13] Lock P, Abram CL, Gibson T, Courtneidge SA (1998). A new method for isolating tyrosine kinase substrates used to identify fish, an SH3 and PX domain-containing protein, and Src substrate.. Embo J.

[14] Abram CL, Seals DF, Pass I, Salinsky D, Maurer L (2003). The adaptor protein fish associates with members of the ADAMs family and localizes to podosomes of Src-transformed cells.. J Biol Chem.

[15] Chan KT, Cortesio CL, Huttenlocher A (2009). FAK alters invadopodia and focal adhesion composition and dynamics to regulate breast cancer invasion.. J Cell Biol.

[16] Oser M, Yamaguchi H, Mader CC, Bravo-Cordero JJ, Arias M (2009). Cortactin regulates cofilin and N-WASp activities to control the stages of invadopodium assembly and maturation.. J Cell Biol.

[17] Seals DF, Azucena EF, Pass I, Tesfay L, Gordon R (2005). The adaptor protein Tks5/Fish is required for podosome formation and function, and for the protease-driven invasion of cancer cells.. Cancer Cell.

[18] Blouw B, Seals DF, Pass I, Diaz B, Courtneidge SA (2008). A role for the podosome/invadopodia scaffold protein Tks5 in tumor growth in vivo.. Eur J Cell Biol.

[19] Stylli SS, Stacey TT, Verhagen AM, Xu SS, Pass I (2009). Nck adaptor proteins link Tks5 to invadopodia actin regulation and ECM degradation.. J Cell Sci.

[20] Robu ME, Larson JD, Nasevicius A, Beiraghi S, Brenner C (2007). p53 activation by knockdown technologies.. PLoS Genet.

[21] Bronner-Fraser M (1995). Origins and developmental potential of the neural crest.. Exp Cell Res.

[22] Stoller JZ, Epstein JA (2005). Cardiac neural crest.. Semin Cell Dev Biol.

[23] Stewart RA, Sanda T, Widlund HR, Zhu S, Swanson KD (2010). Phosphatase-dependent and -independent functions of Shp2 in neural crest cells underlie LEOPARD syndrome pathogenesis.. Dev Cell.

[24] Tobin JL, Di Franco M, Eichers E, May-Simera H, Garcia M (2008). Inhibition of neural crest migration underlies craniofacial dysmorphology and Hirschsprung's disease in Bardet-Biedl syndrome.. Proc Natl Acad Sci U S A.

[25] Epperlein HH, Lofberg J, Olsson L (1996). Neural crest cell migration and pigment pattern formation in urodele amphibians.. Int J Dev Biol.

[26] Kelsh RN, Brand M, Jiang YJ, Heisenberg CP, Lin S (1996). Zebrafish pigmentation mutations and the processes of neural crest development.. Development.

[27] Kucenas S, Takada N, Park HC, Woodruff E, Broadie K (2008). CNS-derived glia ensheath peripheral nerves and mediate motor root development.. Nat Neurosci.

[28] Stylli SS, Stacey TT, Verhagen AM, Xu SS, Pass I (2009). Nck adaptor proteins link Tks5 to invadopodia actin regulation and ECM degradation.. J Cell Sci.

[29] Blake RA, Broome MA, Liu X, Wu J, Gishizky M (2000). SU6656, a selective src family kinase inhibitor, used to probe growth factor signaling.. Mol Cell Biol.

[30] Hanke JH, Gardner JP, Dow RL, Changelian PS, Brissette WH (1996). Discovery of a novel, potent, and Src family-selective tyrosine kinase inhibitor. Study of Lck- and FynT-dependent T cell activation.. J Biol Chem.

[31] Jopling C, den Hertog J (2005). Fyn/Yes and non-canonical Wnt signalling converge on RhoA in vertebrate gastrulation cell movements.. EMBO Rep.

[32] Sharma D, Holets L, Zhang X, Kinsey WH (2005). Role of Fyn kinase in signaling associated with epiboly during zebrafish development.. Dev Biol.

[33] Berzat A, Hall A (2010). Cellular responses to extracellular guidance cues.. EMBO J.

[34] Maurer J, Fuchs S, Jager R, Kurz B, Sommer L (2007). Establishment and controlled differentiation of neural crest stem cell lines using conditional transgenesis.. Differentiation.

[35] Gimona M, Buccione R, Courtneidge SA, Linder S (2008). Assembly and biological role of podosomes and invadopodia.. Curr Opin Cell Biol.

[36] Moustakas A, Heldin CH (2008). Dynamic control of TGF-beta signaling and its links to the cytoskeleton.. FEBS Lett.

[37] Berndt JD, Clay MR, Langenberg T, Halloran MC (2008). Rho-kinase and myosin II affect dynamic neural crest cell behaviors during epithelial to mesenchymal transition in vivo.. Dev Biol.

[38] Herlyn M, Mancianti ML, Jambrosic J, Bolen JB, Koprowski H (1988). Regulatory factors that determine growth and phenotype of normal human melanocytes.. Exp Cell Res.

[39] O'Connor TJ, Neufeld E, Bechberger J, Fujita DJ (1992). pp60c-src in human melanocytes and melanoma cells exhibits elevated specific activity and reduced tyrosine 530 phosphorylation compared to human fibroblast pp60c-src.. Cell Growth Differ.

[40] Tarone G, Cirillo D, Giancotti FG, Comoglio PM, Marchisio PC (1985). Rous sarcoma virus-transformed fibroblasts adhere primarily at discrete protrusions of the ventral membrane called podosomes.. Exp Cell Res.

[41] Erickson CA, Duong TD, Tosney KW (1992). Descriptive and experimental analysis of the dispersion of neural crest cells along the dorsolateral path and their entry into ectoderm in the chick embryo.. Dev Biol.

[42] Valinsky JE, Le Douarin NM (1985). Production of plasminogen activator by migrating cephalic neural crest cells.. Embo J.

[43] Martins-Green M, Erickson CA (1987). Basal lamina is not a barrier to neural crest cell emigration: documentation by TEM and by immunofluorescent and immunogold labelling.. Development.

[44] Raible DW, Wood A, Hodsdon W, Henion PD, Weston JA (1992). Segregation and early dispersal of neural crest cells in the embryonic zebrafish.. Dev Dyn.

[45] Lallier T, Bronner-Fraser M (1992). Alpha 1 beta 1 integrin on neural crest cells recognizes some laminin substrata in a Ca(2+)-independent manner.. J Cell Biol.

[46] Baas D, Malbouyres M, Haftek-Terreau Z, Le Guellec D, Ruggiero F (2009). Craniofacial cartilage morphogenesis requires zebrafish col11a1 activity.. Matrix Biol.

[47] Coyle RC, Latimer A, Jessen JR (2008). Membrane-type 1 matrix metalloproteinase regulates cell migration during zebrafish gastrulation: evidence for an interaction with non-canonical Wnt signaling.. Exp Cell Res.

[48] McCusker C, Cousin H, Neuner R, Alfandari D (2009). Extracellular cleavage of cadherin-11 by ADAM metalloproteases is essential for Xenopus cranial neural crest cell migration.. Mol Biol Cell.

[49] Neuner R, Cousin H, McCusker C, Coyne M, Alfandari D (2009). Xenopus ADAM19 is involved in neural, neural crest and muscle development.. Mech Dev.

[50] Takatsuka A, Yagi R, Koike M, Oneyama C, Nada S (2008). Ablation of Csk in neural crest lineages causes corneal anomaly by deregulating collagen fibril organization and cell motility.. Dev Biol.

[51] Alfandari D, Wolfsberg TG, White JM, DeSimone DW (1997). ADAM 13: a novel ADAM expressed in somitic mesoderm and neural crest cells during Xenopus laevis development.. Dev Biol.

[52] Iqbal Z, Cejudo-Martin P, de Brouwer A, van der Zwaag B, Ruiz-Lozano P (2010). Disruption of the Podosome Adaptor Protein TKS4 (SH3PXD2B) Causes the Skeletal Dysplasia, Eye, and Cardiac Abnormalities of Frank-Ter Haar Syndrome.. American Journal of Human Genetics.

[53] Buschman MD, Bromann PA, Cejudo-Martin P, Wen F, Pass I (2009). The novel adaptor protein Tks4 (SH3PXD2B) is required for functional podosome formation.. Mol Biol Cell.

[54] Gilmour DT, Maischein HM, Nusslein-Volhard C (2002). Migration and function of a glial subtype in the vertebrate peripheral nervous system.. Neuron.

[55] Westerfield M (1993). The Zebrafish Book.

[56] Kimmel CB, Ballard WW, Kimmel SR, Ullmann B, Schilling TF (1995). Stages of embryonic development of the zebrafish.. Dev Dyn.

[57] Stewart RA, Arduini BL, Berghmans S, George RE, Kanki JP (2006). Zebrafish foxd3 is selectively required for neural crest specification, migration and survival.. Dev Biol.

