# Correction: A Src-Tks5 Pathway Is Required for Neural Crest Cell Migration during Embryonic Development

**DOI:** 10.1371/annotation/d66234d8-b722-4b6d-ab8a-73b5f396c37e

**Published:** 2012-08-23

**Authors:** Danielle A. Murphy, Begoña Diaz, Paul A. Bromann, Jeff H. Tsai, Yasuhiko Kawakami, Jochen Maurer, Rodney A. Stewart, Juan Carlos Izpisúa-Belmonte, Sara A. Courtneidge

We have identified inadvertent errors in this manuscript, as described below:

1. The legend to Figure 1D describes the quantification of 3 experiments but in fact is the quantification of one representative experiment of 3 total experiments that were conducted.

2. In Figure 2E, the "Tks5MO + Tks5 RNA" sub-panel is a duplicate of the "Tks5 MO" panel.

3. The legend to Figure 3A is incorrect, and should read "Neural crest specific riboprobes against Sox10, crestin (ctn) and mitf".

4. In Figures 4A and 6B, the images shown are from Tg(FoxD3:GFP) labeled fish, not from Tg(Sox10:RFP) labeled fish as stated.

5. In Figure 4D, the labels for the sub-panels "T5" and "T5MO" were reversed.

Corrected versions of Figures 2 and 4 are provided. We apologize for any confusion our inadvertent errors may have caused, but emphasize that these changes do not affect in any way the conclusions of our studies.

Figure LEGENDS

Figure 2.Decreased Tks5 expression results in neural crest-derived defects.

(A-B) Melanophores within the trunk region above the yolk sac extension in control MO-injected and Tks5 MO-injected embryos were qualitatively (A) and quantitatively (B) analyzed. n=15 embryos and SEM is shown by bar. p values obtained from Student's t-test. ** denotes p <0.01.

(C) Melanophores present in the dorsal, ventral, and lateral pigment lines were quantified to determine degree of murine Tks5 rescue of the decreased pigmentation seen in morphants. Mean values (n=3) and SEM are shown in graph. p values obtained from Student's t-test. ** denotes p <0.01.

(D) Alcian blue staining was performed on indicated embryos to identify craniofacial structures (Meckel's cartilage (mc), palatoquadrate (pq), ceratobranchials (ch), ethmoid plate (ep)). (*) denotes missing structures.

(E) Alcian blue staining was performed on indicated embryos to determine if murine Tks5 could rescue craniofacial defects seen in morphants. Structures were identified as in (D). (*) denotes missing structures.

A revised version of Figure 2 can be seen here: 

**Figure:**
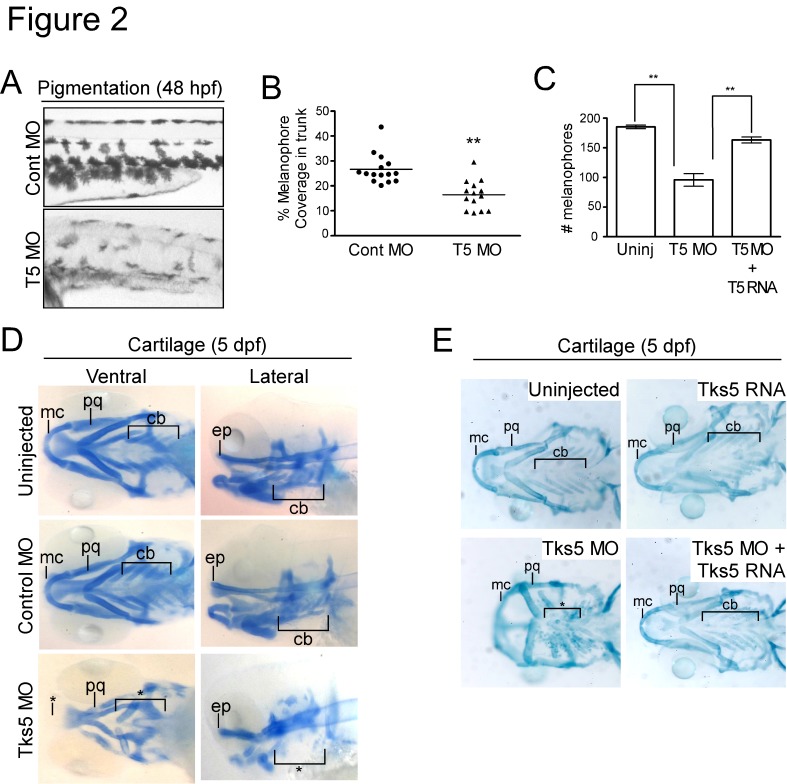


[^]

Figure 4.Neural crest derivatives require a Src-Tks5-dependent pathway in vivo.

(A) Tg(FoxD3:GFP) embryos (8 hpf) were treated with either vehicle (DMSO) or SU6656 for 24 hours and imaged by confocal microscopy to detect neural crest cells. (D= dorsal, V= ventral). Brackets indicate the position of the somites. Scale bar represents 50 mm.

(B-C) Embryos at 15 hpf were treated as indicated for 24 hours and analyzed for pigmentation defects. (B) Embryos where treatment was initiated at 15 hpf were examined for melanophore patterning in the trunk region above the yolk sac extension. (D=dorsal, V=ventral, A=anterior, P=posterior) (C) The total number of melanophores present in the dorsal and ventral pigment lines was counted for embryos within each group as described in Materials and Methods. Mean values (n=3) and SEM were shown in graph. ** denotes p <0.01 for vehicle treated vs. SFK treated comparison.

(D-E) Embryos were injected as indicated and qualitatively analyzed for defects described previously. Morpholino and RNA concentrations detailed in Materials and Methods. (E) Morphants were identified as described in Figure 1D and embryos within each group were quantified (white = morphants, black = normal).

A revised version of Figure 4 can be seen here: 

**Figure:**
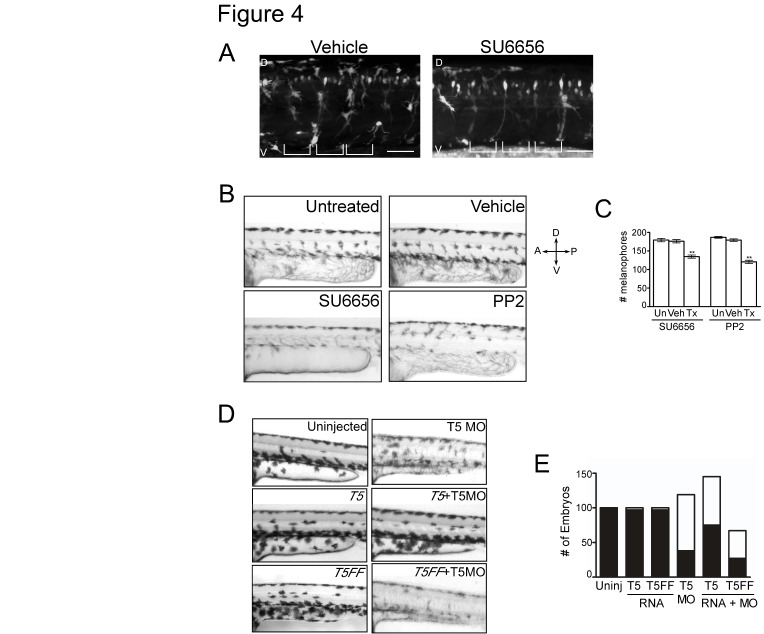


[^] 

