# Supplementary figures and images for: A Src-Tks5 Pathway Is Required for Neural Crest Cell Migration during Embryonic Development
Source: PLoS One. 2011 Jul 25;6(7):e22499. doi: 10.1371/journal.pone.0022499 (PMC3143166; doi:10.1371/journal.pone.0022499)

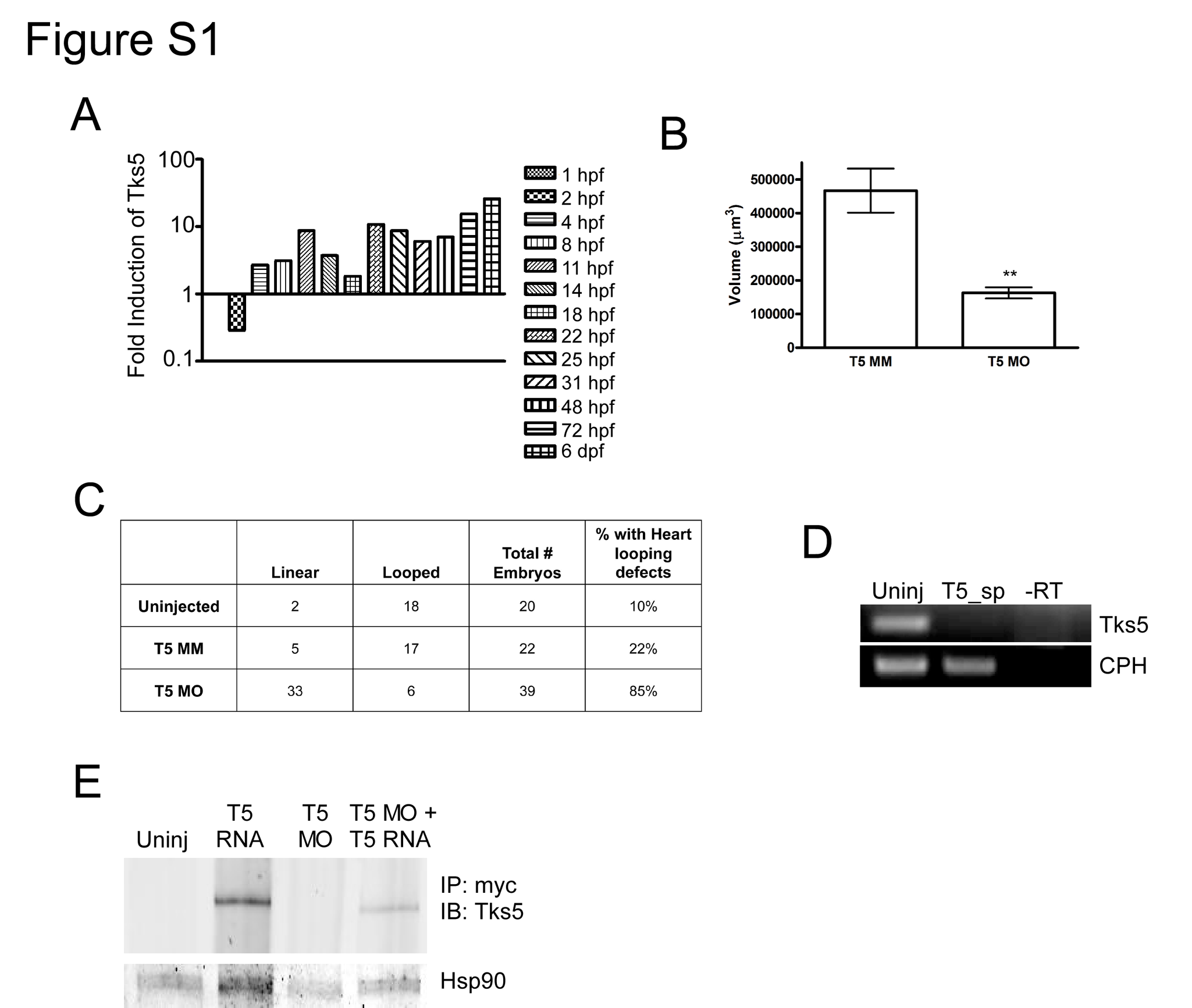

Supplement: Figure S1 — Tks5 is required for embryonic development in Danio rerio . (A) Tks5 expression levels were examined throughout embryonic development in zebrafish by quantitative PCR. Levels were normalized to cyclophilin and fold increase was compared to 4-cell embryonic stage (set at 1). (B) The eye volume (µm3) of control (T5 MM) and Tks5 morphants (T5.1 MO and T5_sp) at 48 hpf was measured as detailed in the Materials and Methods section. Mean values (n = 12 and n = 17, respectively) and SEM are shown. p values obtained from Student's t-test.** denotes p<0.01. (C) Tg (mlc2:GFP) embryos (30hpf) uninjected or injected with either control (T5 MM) or Tks5 morpholinos (T5.1 MO and T5_sp) were examined for the ability to induce heart looping by fluorescence microscopy. Embryos in each group were quantified for the presence of a linear or looped heart. (D) RT-PCR analysis using exon 3 specific primers of uninjected and Tks5_sp MO-injected embryos at 24 hpf. (E) Lysates were obtained from indicated embryos at 24 hpf and immunoprecipitated with anti-Myc antibody followed by immunoblotting for Tks5. The blot was re-probed with anti-Hsp90 antibody for loading controls. (TIF) [file pone.0022499.s001.tif]

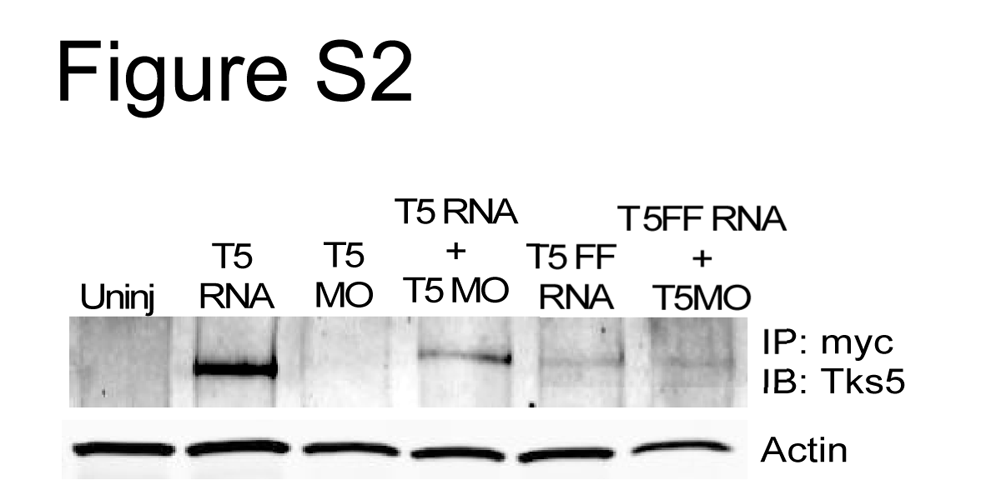

Supplement: Figure S2 — Src phosphorylation of Tks5 affects neural crest-derived cells. Lysates were obtained from indicated embryos (24 hpf) as described in Experimental Procedures. Tks5myc and Tks5FFmyc expression was detected by immunoprecipitation for Myc and immunoblotting for Tks5. Loading was normalized to actin. (TIF) [file pone.0022499.s002.tif]

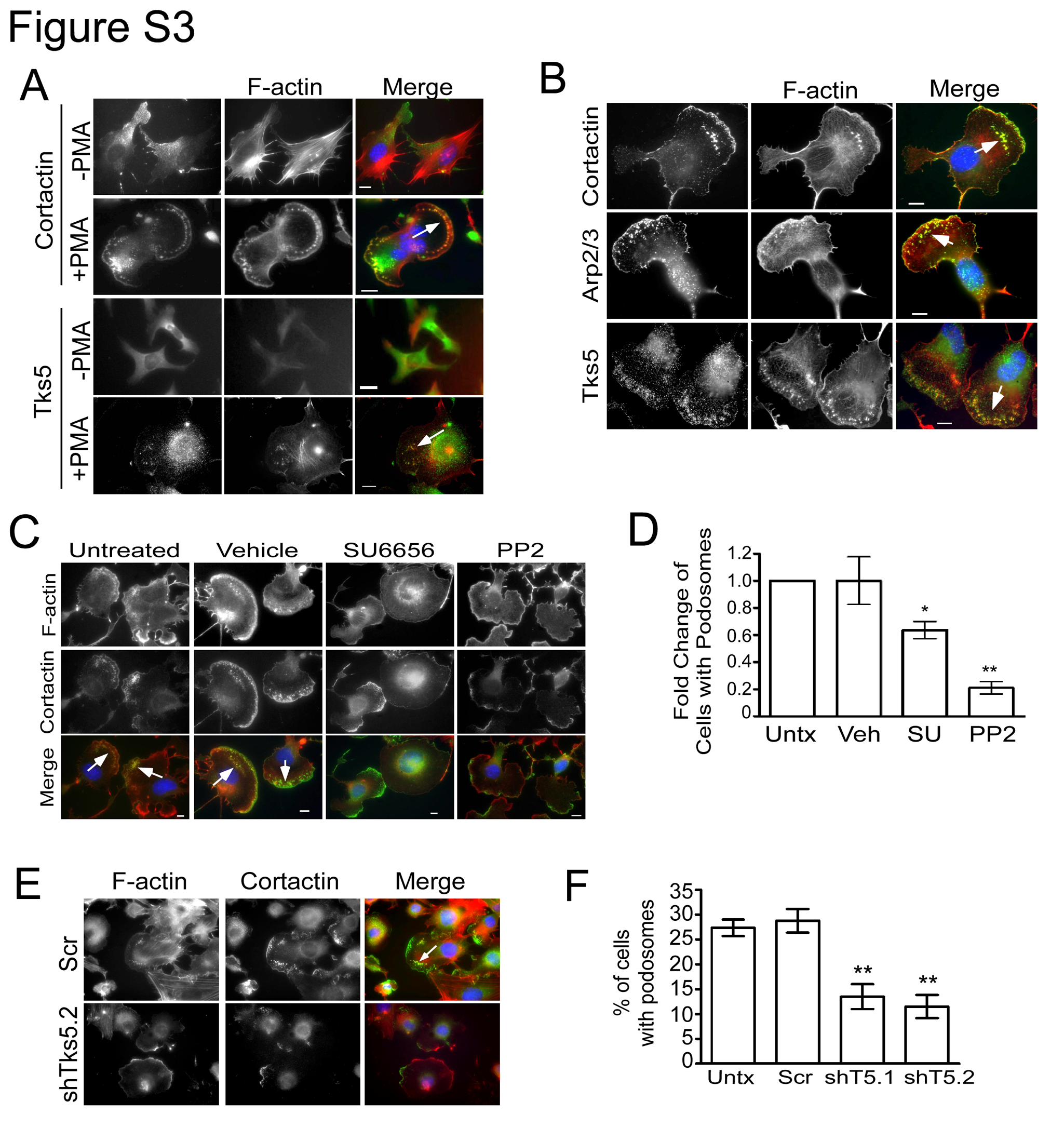

Supplement: Figure S3 — Neural crest stem cells form podosomes. (A) JOMA1.3 cells were stained for F-actin (phalloidin) and either cortactin or Tks5 in the presence and absence of PMA (25 ng/ml) to identify formation of podosomes (arrow). (B) PMA-stimulated (25 ng/ml) JOMA1.3 cells were immunostained for F-actin (using phalloidin) and the podosome markers cortactin, Arp2/3, and Tks5. (C–D) Vehicle (DMSO) or SFK inhibitors (SU6656 and PP2) were added to JOMA1.3 cells prior to PMA stimulation. (C) Analysis of podosome formation was conducted by immunostaining for F-actin (phalloidin) and cortactin (arrows). (D) The total number of cells with podosomes was quantified for each treatment group and analyzed as fold change of cells with podosomes compared to untreated cells. Mean values (n = 3) and SEM were shown in graph. p values obtained from Student's t-test. * denotes p<0.05 for untreated vs. SFK treated comparison; ** denotes p<0.01 for untreated vs. SFK treated comparison. (E–F) PMA-treated control (uninfected and scrambled shRNA) and Tks5 knockdown (shT5.1, and shT5.2) cells were stained for F-actin (phalloidin) and cortactin to identify formation of podosomes. (F) Percentage of cells possessing podosomes was calculated as previously described. Mean values (n = 3) and SEM are shown in graph. ** denotes p<0.01 for scrambled versus shT5.1 and shT5.2. p values obtained from Student's t-test. In all cases, the white arrows point to clusters of podosomes. (TIF) [file pone.0022499.s003.tif]
